# A study on the effect of 12-week unstable bench press barbell training on improving upper limb muscle strength of college students

**DOI:** 10.3389/fphys.2026.1826652

**Published:** 2026-06-30

**Authors:** Shuai Zhu, Anting She, Haifeng Tao

**Affiliations:** 1 Xinjiang University, Urumqi, China; 2 Tongji University, Shanghai, China

**Keywords:** agonist activation level, antagonist co-activation, bench press exercise, MVIC, strength quality, surface electromyography signal, unstable resistance training

## Abstract

This study aimed to explore the effect of 12-week unstable bench press barbell training on improving upper limb muscle strength in college students and analyze the potential mechanisms of strength changes induced by training. Eighteen healthy male college student volunteers with no upper limb strength training experience were randomly divided into an unstable bench press training group (IBP group, n=9) and a Smith machine bench press training group (SBP group, n=9). The two groups received 3 sessions of bench press training per week for 12 consecutive weeks under unstable and stable conditions, respectively. The IBP group trained with 55%→65%→75% 1RM across three 4-week phases, while the SBP group trained with 65%→75%→85% 1RM, reflecting the greater coordinative and postural demands imposed by the unstable condition. The rationale for using lower nominal loads in the unstable condition was that instability inherently increases neuromuscular and postural control demands, thereby equating the effective physiological training stimulus between conditions. Before and after the training, indicators including upper arm muscle circumference, 1 repetition maximum (1RM), maximal voluntary isometric contraction (MVIC), unstable bench press movement stability were tested, and surface electromyography (sEMG) signals of biceps brachii (BB), triceps brachii (TB), anterior deltoid (AD), posterior deltoid (PD) and pectoralis major (PM) were collected during the unstable bench press test. The results showed that only the SBP group had a significant increase in relaxed upper arm muscle circumference after training (P<0.05). Both groups achieved significant improvements in 1RM and MVIC after 12 weeks of training (P<0.05). Group × time interaction was significant for 1RM (F = 4.36, p=0.033, d=0.82, 95% CI = 1.24 to 18.67), indicating that IBP improved significantly more than SBP. For MVIC, the group × time interaction was not significant (F = 0.06, p=0.809), suggesting comparable static strength gains between groups. The acceleration integral value of the IBP group on the X-axis in the unstable bench press test was significantly smaller than that of the SBP group, with significant group × time interactions on the X-axis (p=0.037) and Y-axis (p=0.049). In the post-training unstable bench press test, the integrated electromyography (iEMG) of TB, AD, PD and PM in the IBP group and TB, AD in the SBP group decreased significantly (P<0.05), and only the PD-PM co-activation ratio in the IBP group decreased significantly after training (P<0.05). In the Smith machine bench press fatigue test, the IBP group completed significantly more repetitions than the SBP group after training (p<0.05). It is concluded that both 12-week unstable and stable bench press barbell training can significantly improve the upper limb muscle strength of college students, and unstable bench press training demonstrates advantages over stable Smith machine training for dynamic strength and movement stability. Unstable bench press training may improve agonist contraction ability and muscle work efficiency, accompanied by plausible neuromuscular adaptations in coordination and antagonist muscle regulation. However, interpretations should be tempered by the task-specific testing design, which may favor the unstable training group.

## 1 Introduction

Strength quality refers to the comprehensive ability to produce force effectively under specific movement constraints, including maximal strength, explosive force output, and neuromuscular coordination. Strength quality is a core component of physical fitness, which improves athletic performance in competitive sports, promotes overall physical health, maintains high-quality daily life, and ensures efficient completion of daily activities (Folland and Williams, 2007). Upper limb muscle strength is closely associated with performance of daily tasks, heavy physical labor, whole-body coordination, postural stability, and reduced risk of musculoskeletal injuries (Ostrowski et al., 2017). However, the national student physical health monitoring data show that poor upper limb muscle strength has become a common problem among college and middle school students in China (Anderson and Behm, 2004). Therefore, exploring an effective training method to improve upper limb muscle strength has important practical and public health significance (Behm and Anderson, 2006).

Resistance training is a recognized method to improve muscle strength (Behm et al., 2010). In recent decades, unstable resistance training (URT) has been increasingly adopted in fitness and competitive sports. Early studies on unstable resistance training date back to the 1990s and early 2000s. Controllable instability increases neuromuscular load, enhances activation of deep trunk stabilizers and core muscles, and improves postural control and balance (Behm et al., 2005). Compared with stable resistance training, URT may optimize the coordination between agonist and antagonist muscles by reducing excessive antagonist co-contraction during the concentric phase, thereby promoting more favorable neuromuscular adaptability, which refers to the capacity of the nervous system to optimize muscle recruitment and coordination in response to training (Costello, 2022). However, some studies hold that the inherent instability of unstable resistance training may affect force generation and exercise efficiency due to excessive co-activation or energy dissipation, thus limiting its effect compared with traditional stable training programs (Dunnick et al., 2015). Indeed, the existing literature presents conflicting findings: while some investigations report superior strength and neuromuscular adaptations following unstable training (Behm et al., 2005; Costello, 2022), others have found neutral or even negative effects on maximal force production when compared with stable resistance training (Dunnick et al., 2015; Unhjem et al., 2016; Sanchez-Sanchez et al., 2022). These discrepancies may stem from differences in the type and magnitude of instability, the training protocols employed, the participant characteristics, and the specificity of testing procedures. A systematic comparison and reconciliation of these conflicting findings is currently lacking. Up to now, the effect of unstable resistance training on improving upper limb muscle strength has not been concluded, which highlights the necessity of further empirical research (Rodriguez-Ridao et al., 2020).

Barbell bench press is a widely used upper-body resistance training exercise (Schick et al., 2010). This study selected unstable load bench press, a specific form of unstable resistance training (URT), and Smith machine bench press as training interventions. This study examined differences in muscle circumference, one-repetition maximum (1RM), maximal voluntary isometric contraction (MVIC), triaxial acceleration, and electromyography signals after 12 weeks of intervention. We hypothesized that unstable resistance training would induce greater improvements in 1RM, MVIC, movement stability, and neuromuscular coordination, along with reduced antagonist co-activation compared with stable training.

## 2 Materials and methods

### 2.1 Study design

**Figure 1:**
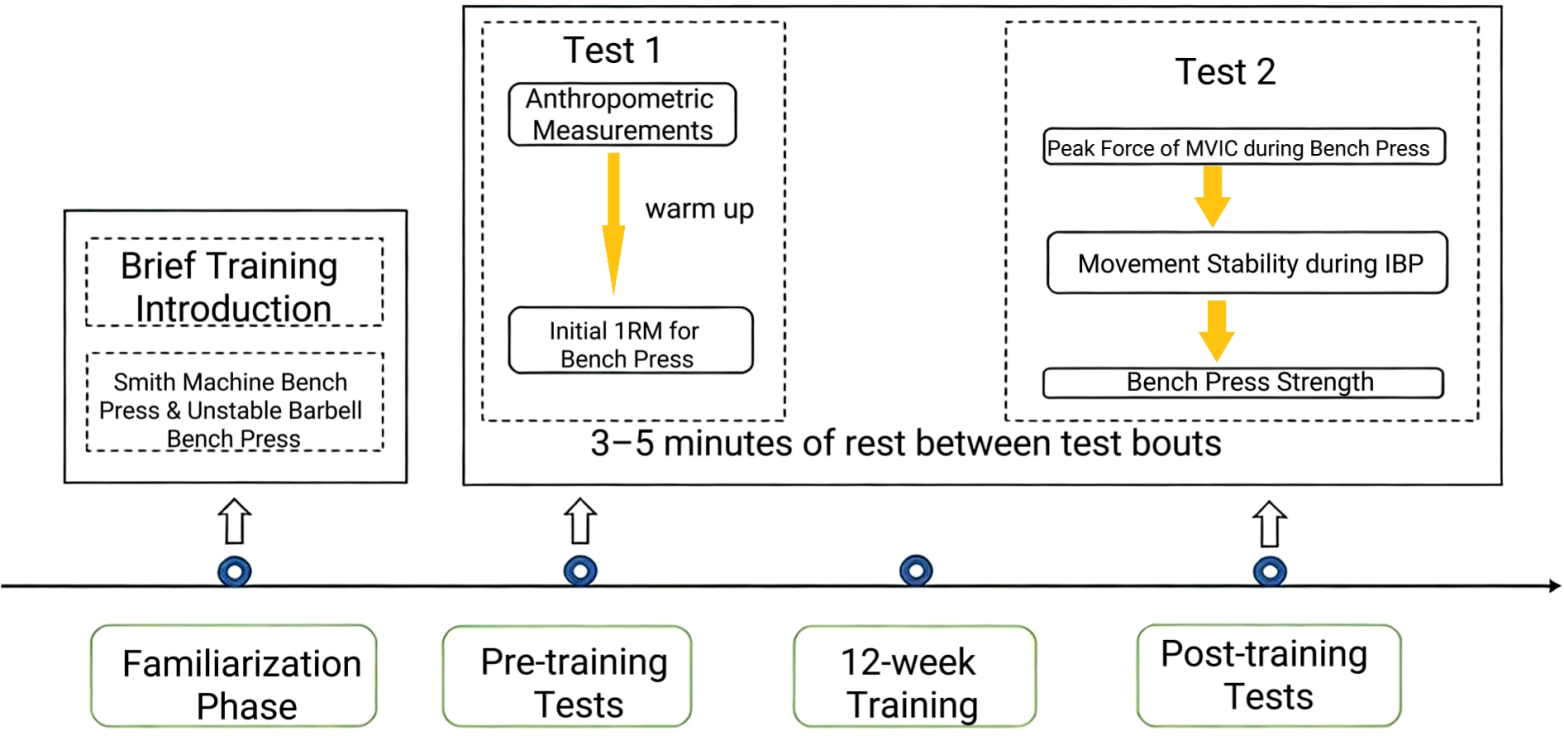
Overall experimental design. IBP, unstable bench press group; SBP, Smith machine bench press group; 1RM, one-repetition maximum; MVIC, maximal voluntary isometric contraction; sEMG, surface electromyography.

The experiment consisted of four stages: familiarization stage, pre-training test, training intervention and post-training test (**Figure 1**). All participants completed a 2-week familiarization period with unstable barbell bench press before random group assignment. The two groups of subjects followed the bench press resistance training program for 12 weeks under stable and unstable conditions, respectively. Pre-training test (Test 1) and post-training test (Test 2) were conducted both before and after the training intervention, with an interval of 48 hours between the two tests.

In Test 1, subjects completed upper arm circumference measurement, MVIC testing, and 1RM testing. In Test 2, subjects completed MVIC testing, two types of bench press movement tests, and a Smith machine bench press fatigue test. Surface electromyography (sEMG) signals and triaxial acceleration sensor data were recorded during the entire bench press movement test. Both tests were conducted at the same time on different days, with 3–5 minutes of rest between test items to reduce fatigue interference.

### 2.2 Subjects

*A priori* sample size calculation was completed by G*Power 3.1.9.7, with α=0.05, power=0.95, effect size f=0.80, and the main outcome indicator was 1RM, so the sample size of each group was determined to be 8 cases. Considering the potential of sample loss during the experiment, 22 subjects were initially recruited. The subjects were divided into two groups according to the pre-test 1RM, and then the training method was determined by random drawing. A total of 22 participants were recruited, 18 completed the experiment, and 4 withdrew due to time conflict. Finally, 18 subjects completed the experiment, including 9 in the unstable bench press group (IBP) and 9 in the Smith machine bench press group (SBP).

All subjects had no history of upper limb musculoskeletal injuries in the past six months, no experience in upper limb system strength and special ability training, and no special upper limb strength training every day except daily physical activities. Subjects were healthy, without neurological diseases, with moderate body shape (BMI 18.5–24.0) and low subcutaneous fat confirmed by visual assessment. They understood the experimental process, purpose and existing risks before the experiment and participated voluntarily. The basic characteristics of the participants are shown in **Table 1**.

**Table 1:** Basic characteristics of participants (n=18).

Group	number of people	Age (yr)	Height (cm)	Weight (kg)
SBP	9	18.78 ± 0.83	174.78 ± 6.82	62.50 ± 10.40
IBP	9	19.11 ± 0.60	175.11 ± 3.30	65.22 ± 6.210

Values are presented as mean ± SD.

IBP, unstable bench press group; SBP, Smith machine bench press group.

### 2.3 Training intervention protocol

Both groups received 3 bench press training sessions per week for 12 weeks, with 5 sets per session and 6 repetitions per set. Load intensities were set based on progressive overload guidelines for untrained individuals. Moderate-intensity resistance training (e.g., 65–75% 1RM, 5 sets × 6 repetitions) has been demonstrated to effectively stimulate maximal strength gains in untrained individuals when coupled with appropriate volume and effort (Kidgell et al., 2011; Radaelli et al., 2015; Damas et al., 2019). While higher loads (≥85% 1RM) are generally recommended for maximal strength development in trained individuals, the 5 × 6 protocol at moderate intensities provides sufficient mechanical tension and metabolic stress to drive neuromuscular adaptations in novice populations (Behm et al., 2010; Kidgell et al., 2011).Moderate intensity can effectively stimulate initial adaptation; unstable conditions further increase task difficulty: the IBP group adopted 55% 1RM for weeks 1-4, 65% 1RM for weeks 5-8, and 75% 1RM for weeks 9-12, with an interval of 1-1.5 minutes between sets; the SBP group adopted 65% 1RM for weeks 1-4, 75% 1RM for weeks 5-8, and 85% 1RM for weeks 9-12, with the same interval time as the IBP group.

The unstable training group adopted a relatively lower load (55–75% 1RM) because the unstable environment increases postural control and neuromuscular load, while the stable training group used a higher load (65–85% 1RM) to maintain equivalent effective training intensity. This intensity-equivalence rationale is supported by evidence that unstable conditions increase the coordinative demands and neuromuscular activation required to execute the same movement, effectively elevating the physiological challenge at a given nominal load (Behm et al., 2002; Behm et al., 2005). Specifically, performing bench press with elastic band-induced instability requires additional motor unit recruitment to stabilize the barbell, which compensates for the lower absolute load. Pilot testing from our laboratory (n=6) confirmed that perceived exertion (RPE) was comparable between the IBP group training at 55% 1RM and the SBP group training at 65% 1RM during the initial training phase (mean RPE: 14.2 ± 1.3 vs. 13.8 ± 1.5, respectively). Additionally, training adherence exceeded 90% in both groups (IBP: 93.5%; SBP: 94.2%), and the actual completed session loads (sets × reps × %1RM) were documented to confirm protocol fidelity.

In the unstable bench press, elastic bands with dangling weights were attached to both ends of the barbell to create local dynamic instability. The IBP group used a comprehensive training rack (PowerLift, USA), and two elastic bands (Decathlon, France) were fixed to both ends of the barbell with small weight plates dangling vertically to create dynamic local instability. The SBP group used a traditional Smith machine (Magnum Smith Machine, Matrix Fitness, China) for training.

Several methodological limitations exist when comparing factor loadings across different groups. It should be noted that despite the physiological rationale for differential loading, the two interventions were not load-matched in absolute or relative terms. As such, direct between-group comparisons of strength outcomes should be interpreted with caution, and the differential loading scheme should be considered when evaluating the relative efficacy of each training modality.

### 2.4 Test indicators

#### 2.4.1 Muscle strength indicators

1RM was used as the indicator of dynamic maximum muscle strength, and the test was carried out in accordance with the maximum strength evaluation guidelines of the National Strength and Conditioning Association (NSCA) (Latash, 2018). To reduce learning effects in untrained participants, all subjects completed standardized familiarization before formal 1RM testing. The 1RM test was performed using the Smith machine to ensure consistency across groups. Before the test, the subjects conducted self-warm-up with a load that could complete 6–10 repetitions (about 50% of the predicted 1RM). After a 1–5 minute rest, the subjects selected the weight according to the initial attempt, which could complete 3 repetitions (about 80% of the predicted 1RM). Then, the resistance was gradually increased by 5%-10% for each attempt, and the 1RM was determined within 5 attempts, with a 3–5 minute rest between each attempt.

MVIC of bench press was used as the indicator of static maximum muscle strength (Psek and Cafarelli, 1993). The subjects took the MVIC test one week before the start and one week after the end of the training, which was arranged 48 hours after the 1RM test. Post-tests were performed one week after training to avoid acute fatigue and reflect stable chronic adaptations. The subjects performed the bench press on the Smith machine with the common bench press method: chest out and abdomen in, hips and knees bent, feet firmly on the ground, back as close to the flat plate as possible, grip width equal to shoulder width, barbell moving up and down vertically at the position of the middle chest, wrists kept straight, elbows abducted as much as possible but not exceeding the shoulders, and barbell lowered as much as possible but not placed on the chest. Before the test, the barbell bar was lifted to the position where the subjects’ hands were slightly bent, the static MVIC tester was placed on the left side of the barbell bar, and the barbell bar, dynamometer and weight were connected with an iron chain. A calibrated digital dynamometer was fixed on the left side of the barbell and connected by a steel chain to measure maximum isometric force. After the test command was issued, the dynamometer value was observed, and the test was ended when the test instrument gave a prompt of maximum force, and the electromyography information was recorded synchronously.

#### 2.4.2 Movement stability test

The subjects performed 5 repetitions of unstable bench press with a load equivalent to 60% of their own 1RM, and the movement rhythm was consistent with the training rhythm. A Kinv TS three-axis accelerometer (STT-isen, Spain, Model IWS) was used to measure the oscillation and stability of the barbell during the movement. The device was fixed on the right edge of the barbell, and the X, Y and Z axes of the three-axis acceleration corresponded to the frontal axis, vertical axis and sagittal axis of the subject, respectively (Carolan and Cafarelli, 1992). Before the test, the acceleration was calibrated along all three axes, and all three-axis acceleration data were sampled continuously at 100Hz. The recording of acceleration signals was synchronized with the electromyography test.

The acceleration signals recorded during the rest period were excluded, and the corresponding data of each bench press repetition were extracted. According to the method of previous studies (Marcos-Frutos et al., 2025), a fourth-order Butterworth filter was used to filter the three-axis (X/Y/Z) acceleration data (Farina et al., 2014). After filtering, the acceleration signals of the unstable bench press test were full-wave rectified. Finally, the average acceleration amplitude of each axis was calculated to quantify the movement stability during the unstable bench press.

#### 2.4.3 Unstable bench press barbell movement test

Referring to previous relevant studies (Wang et al., 2022), barbell plates were connected to both ends of the barbell through elastic bands to generate unstable load during the bench press barbell movement. The mass of the barbell bar used in the experiment was 6 kg, and the subjects determined the mass of the barbell plates connected to both ends of the elastic band according to the load standard of 60% 1RM of their own bench press.

During the test, the subjects lay supine on the bench press bench, with their head, upper back and buttocks close to the bench surface, feet naturally on the ground with shoulder width; hands grasped the barbell bar with a closed pronated grip, and the grip width was a normal grip width slightly wider than the shoulder (Li et al., 2025). Under the full protection of the spotters, the subjects lifted the barbell vertically from the bench press rack until the arms were fully extended, and stabilized the barbell directly above the chest; then completed the bench press movement as required, with the time of barbell lifting and lowering controlled at 3 s respectively, and a 2 s rest was set between two consecutive bench press movements. During the bench press movement test, the subjects were instructed to try their best to maintain the stability of the barbell movement, and verbal encouragement was given to ensure that the subjects made their best efforts to complete the maximum number of bench press barbell repetitions.

#### 2.4.4 Surface electromyography signal collection

According to previously published research reports (Zemková et al., 2021), sEMG electrodes were attached to the midpoint of the muscle belly of the right BB, TB, AD, PD and upper PM of the subjects with an interval of 2 cm. A total of five muscles were assessed: biceps brachii (BB), triceps brachii (TB), anterior deltoid (AD), posterior deltoid (PD), and pectoralis major (PM).Before electrode attachment, the corresponding skin parts were standardized treated by shaving, light grinding with fine sandpaper and wiping with alcohol wipes to ensure good electrical contact between the electrodes and the skin and reduce the contact resistance between the electrodes.

A wireless electromyography test system BTS FREEEMG 1000 produced by BTS Company (Garbagnate Milanese MI, Italy) was used to collect sEMG signals with a sampling frequency set at 1000 Hz (Golas et al., 2017). The collected original sEMG signals were preprocessed: a fourth-order Butterworth filter was used to implement 5~500 Hz band-pass filtering, and the zero-phase shift method was selected in the filtering process to avoid signal phase distortion (Zemková and Zapletalová, 2022); the filtered signals were full-wave rectified, and the root mean square (RMS) value was calculated through a 50 ms time sliding window to extract the sEMG signal envelope.

RMS represents the instantaneous muscle activation level computed over a 50-ms sliding window and was used as the primary variable for assessing activation amplitude during the dynamic bench press movement. The integrated EMG (iEMG) was calculated as the integral of the full-wave rectified EMG signal across the entire concentric phase, representing the total neuromuscular activation within that movement cycle. The concentric phase was operationally identified as the period during which the barbell moved vertically upward (against gravity) from the chest to full elbow extension, confirmed through synchronized accelerometer data (Z-axis). The iEMG values were normalized to the maximal voluntary contraction (MVC) iEMG obtained during the MVIC test (%MVC). The co-activation ratio was defined as: antagonist iEMG (normalized)/agonist iEMG (normalized) during the concentric phase. For each muscle pair, the antagonist muscle was defined as the muscle opposing the primary action: BB as antagonist to TB (elbow extension), AD and PD as antagonists to PM and TB (shoulder horizontal adduction and extension). All EMG analyses were performed using custom scripts in MATLAB (version R2020a, MathWorks, USA).

To objectively evaluate the neuromuscular work efficiency, the RMS value of electromyography recorded during the entire bench press process was standardized with the electromyography level of maximal voluntary contraction (MVC) (Huang et al., 2014), so as to reflect the actual muscle activation intensity of agonist and antagonist muscles during the movement.

## 3 Results

### 3.1 1RM and MVIC

**Figure 2:**
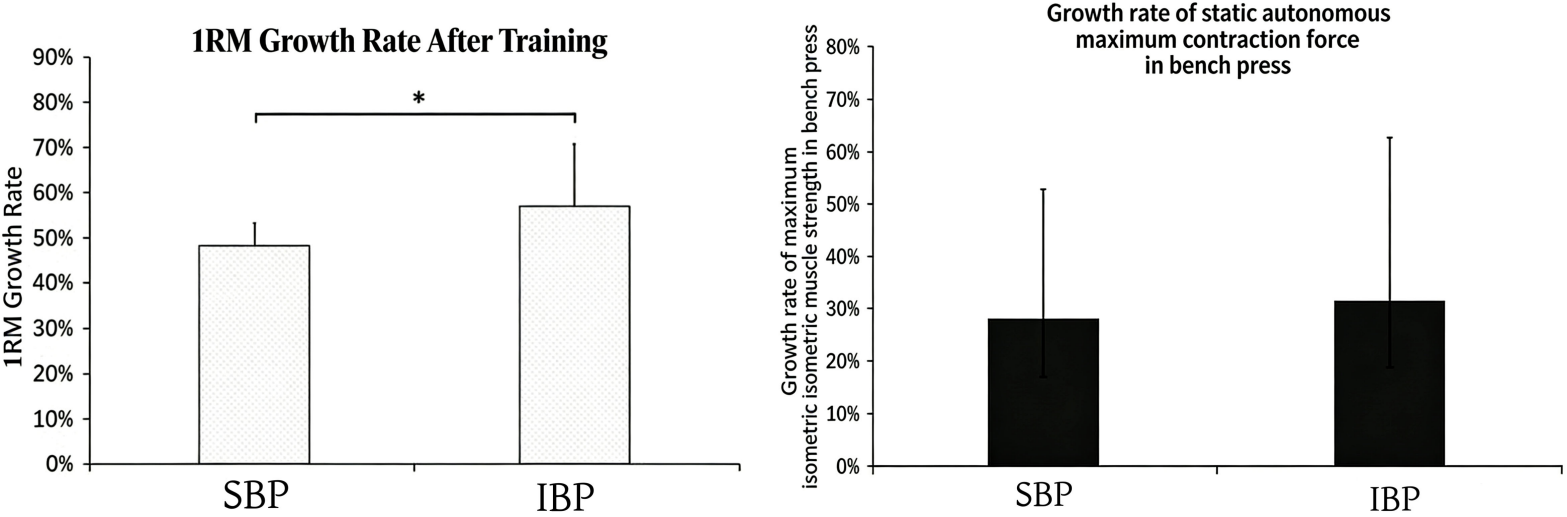
Changes in average and standard deviation of bench press 1RM and MVIC peak force before and after training in IBP and SBP groups. IBP, unstable bench press group; SBP, Smith machine bench press group; 1RM, one-repetition maximum; MVIC, maximal voluntary isometric contraction; *p < 0.05 for within-group pre-post comparisons. Data are presented as mean ± SD.

As shown in **Table 2** and **Figure 2**, after 12 weeks of progressive load bench press training, the 1RM values of both the IBP group and the SBP group increased significantly (P<0.001; P<0.001), while there was no significant difference in the 1RM values between the two groups before and after the training intervention (P = 0.196; P = 0.683). Group × time interaction was significant for 1RM (F = 4.36, p=0.033, Cohen’s d=0.82, 95% CI = 1.24 to 18.67 kg), confirming that the improvement in the IBP group was significantly greater than that in the SBP group. As shown in **Figure 2a**, the increase rate of 1RM in the IBP group (47.38% ± 15.82%) was significantly different from that in the SBP group (33.54% ± 5.85%) (P = 0.033<0.05).

**Table 2:** Comparison of 1RM and MVIC before and after training in IBP and SBP groups (kg, M ± SD).

Group	1RM pre-trainig (kg)	1RM post-training (kg)	MVIC pre-training (kg)	MVIC post-training (kg)
IBP	42.34 ± 4.78	62.36 ± 9.30*	97.92 ± 28.00	124.37 ± 23.69*
SBP	45.58 ± 5.09	60.74 ± 6.13*	91.76 ± 19.52	113.94 ± 12.27*

IBP, unstable bench press group; SBP, Smith machine bench press group. * p<0.05 for within-group pre-post comparison. 1RM, one-repetition maximum; MVIC, maximal voluntary isometric contraction.

* indicates that there is a significant difference in the results of the same training group before and after the intervention.

The results of MVIC test of the two groups before and after the training intervention (**Table 2**) showed that the MVIC of both the IBP group and the SBP group increased significantly after 12 weeks of training (P = 0.009; P = 0.006), while there was no significant difference in MVIC between the two groups before and after the training (P = 0.5326; P = 0.261). The group × time interaction for MVIC was not significant (F = 0.06, p=0.809, d=0.12, 95% CI=-12.35 to 15.76 kg), indicating that the two training modalities produced comparable improvements in maximal isometric strength. As shown in **Figure 2b**, there was no significant difference in the increase rate of bench press MVIC between the IBP group (31.19% ± 31.23%) and the SBP group (27.94% ± 24.64%) (P = 0.809>0.05).

Group × time interaction was significant for 1RM (F = 4.36, p=0.033, d=0.82, 95% CI = 1.24 to 18.67), indicating that the improvement in the IBP group was significantly greater than that in the SBP group.

### 3.2 Upper arm circumference measurement results

As shown in **Table 3**, there was no significant difference in the relaxed upper arm muscle circumference and maximum upper arm muscle circumference of the IBP group before and after the training intervention (P = 0.057; P = 0.881, both P>0.05). The relaxed upper arm muscle circumference of the SBP group had a significant difference before and after the training intervention (P = 0.007<0.05), while the maximum upper arm muscle circumference had no significant difference (P = 0.263).

**Table 3:** Comparison of relaxed and maximal upper arm circumference in IBP and SBP groups (cm, M ± SD).

Group	Relaxed arm circumference pre-training (cm)	Relaxed arm circumference post-training (cm)	Maximal arm circumference pre-training (cm)	Maximal arm circumference post-training (cm)
IBP	27.01 ± 2.70	27.36 ± 2.74	32.71 ± 1.59	32.62 ± 2.62
SBP	25.48 ± 2.33	26.10 ± 2.76*	31.32 ± 2.01	32.13 ± 1.78

IBP, unstable bench press group; SBP, Smith machine bench press group. * p<0.05 for within-group pre-post comparison.

* indicates that there is a significant difference in the results of the same training group before and after the intervention.

The pre-experiment measurement found that there was no significant difference in the relaxed and maximum upper arm muscle circumference between the IBP group and the SBP group, and there was also no significant difference in the two muscle circumferences between the two groups after the training.

### 3.3 Movement stability of unstable bench press training

**Figure 3:**
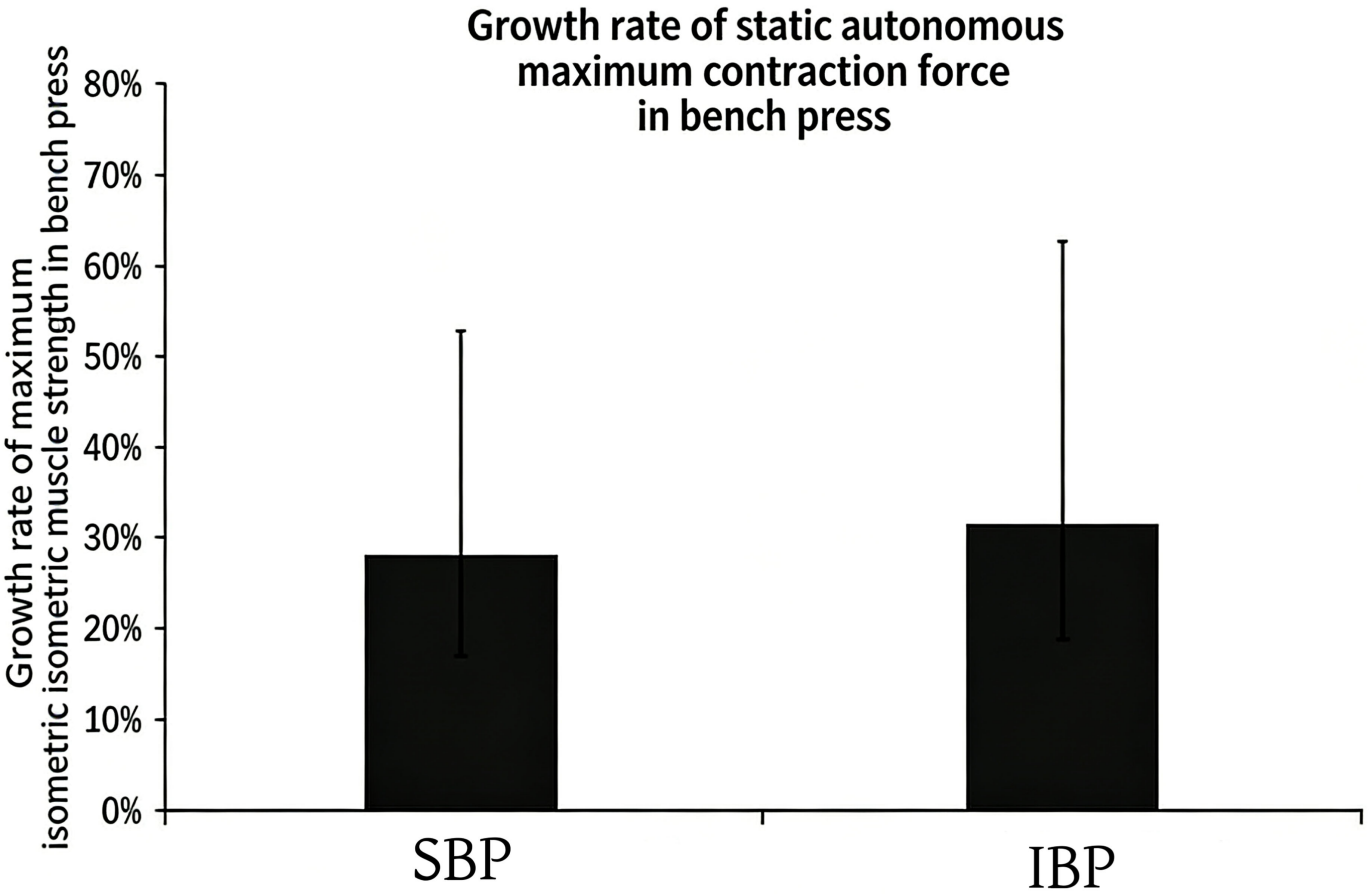
Comparison of amplitude of IBP group and SBP group on X, Y and Z axes before and after training intervention. Data are presented as mean ± SD. IBP, unstable bench press group; SBP, Smith machine bench press group; *p<0.05 for within-group pre-post comparison.

The measured integral acceleration amplitude of the IBP group and SBP group during unstable bench press with 60% 1RM is shown in **Figure 3**. The results of repeated measures analysis of variance showed that training time had a significant main effect on X-axis acceleration (p<0.05), a marginally significant main effect on Y-axis acceleration (p=0.061), and no significant main effect on Z-axis acceleration (p=0.498). The interaction effect of training time × group on X-axis and Y-axis acceleration was significant (X-axis: F = 4.18, p=0.037, d=0.81; Y-axis: F = 3.92, p=0.049, d=0.74), while there was no interaction effect on Z-axis (p=0.124).

The results found that compared with before training, the integral acceleration amplitude of X-axis, Y-axis and Z-axis of both the IBP group and the SBP group showed a downward trend after training. Specifically, the decrease rate of acceleration amplitude of the IBP group was significantly higher than that of the SBP group, among which the difference in the decrease range of X-axis was the most obvious (IBP group: 25.24% ± 15.99%, SBP group: 1.93% ± 37.07%). In contrast, the decrease of integral acceleration amplitude of the IBP group reached a significant level only on the X-axis and Y-axis, and the decrease on the Z-axis did not reach a significant level; the integral acceleration amplitude of the SBP group on each axis before and after training had no statistically significant change.

### 3.4 Agonist activation level

**Figure 4:**
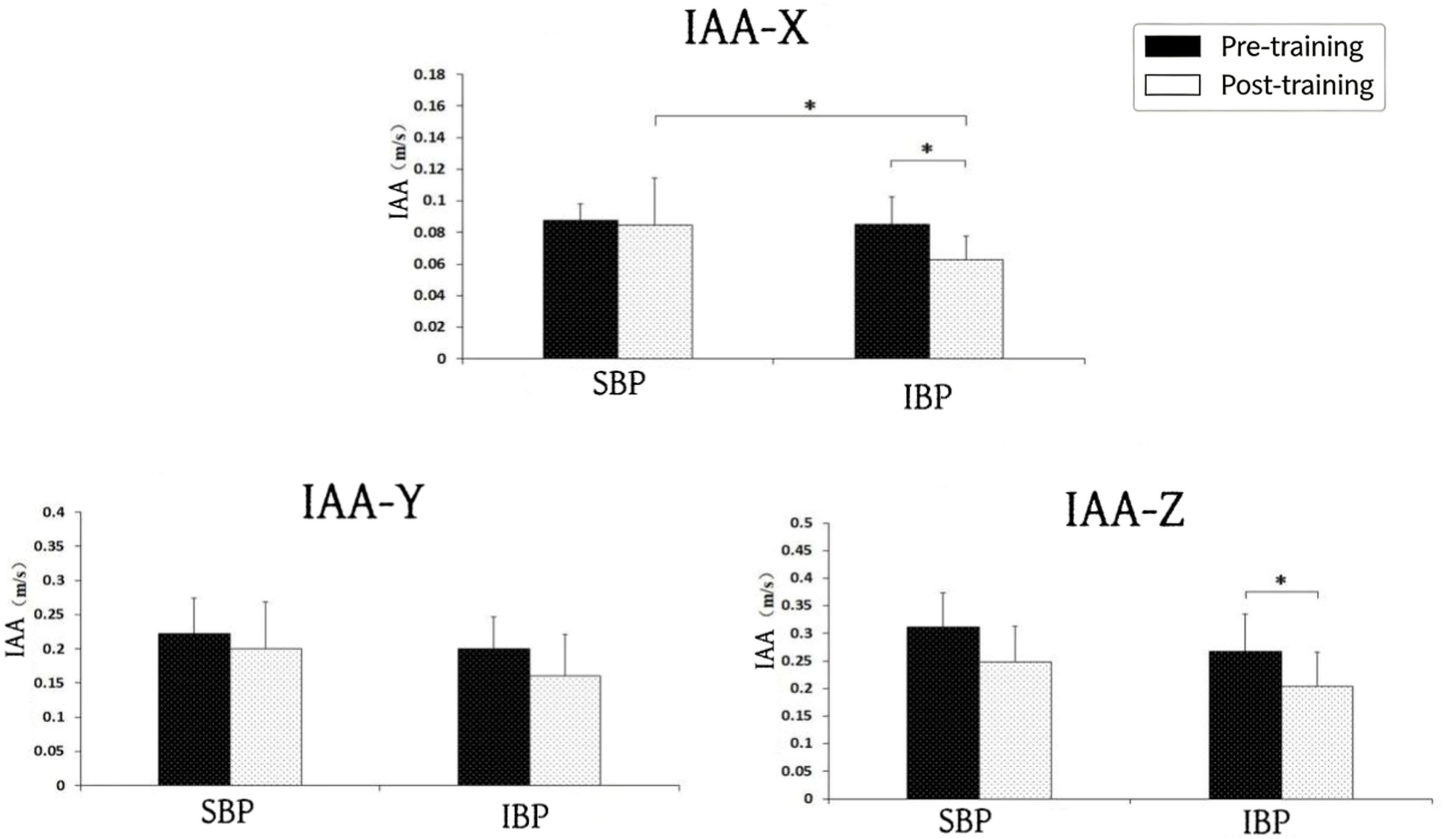
Pre- and post-training comparison of main muscle iEMG in IBP test for SBP and IBP groups. IBP, unstable bench press group; SBP, Smith machine bench press group; BB, biceps brachii; TB, triceps brachii; AD, anterior deltoid; PD, posterior deltoid; PM, pectoralis major; iEMG, integrated electromyography. *p<0.05 for within-group pre-post comparison.

As shown in **Table 4** and **Figure 4**, except for the increase of iEMG of BB in the SBP group, the iEMG amplitude of other major muscles in both groups decreased to different degrees after training. There was no significant difference in each muscle between the two groups before and after training. The iEMG of major muscles in the two groups was at a similar level before training (Pre-training: BB: P = 0.346; TB: P = 0.436; AD: P = 0.699; PD: P = 0.699; PM: P = 0.979). Although the iEMG of major muscles in the two groups decreased by different amplitudes after training, there was no significant difference after test (Post-training: BB: P = 0.068; TB: P = 0.124; AD: P = 0.364; PD: P = 0.147; PM: P = 0.156).

**Table 4:** Percentage changes in integrated electromyography (iEMG) of main muscles in IBP test for IBP and SBP groups (%, M ± SD).

Muscle	IBP	SBP
Biceps Brachii (BB)	-25.71 ± 52.38	22.67 ± 118.39
Triceps Brachii (TB)	-49.79 ± 41.78	-35.06 ± 30.55
Anterior Deltoid (AD)	-62.61 ± 22.92	-49.70 ± 22.62
Posterior Deltoid (PD)	-45.12 ± 34.66	-22.47 ± 47.91
Pectoralis Major (PM)	-41.07 ± 44.62	-5.93 ± 84.89

IBP, unstable bench press group; SBP, Smith machine bench press group. * p<0.05 for within-group pre-post comparison.

Except for BB in the IBP group (BB: P = 0.260), the iEMG of TB, AD, PD and PM decreased significantly after training (TB: P = 0.021; AD: P = 0.008; PD: P = 0.007; PM: P = 0.038). The number of muscles with decreased iEMG in the IBP group was more than that in the SBP group in the unstable test.

### 3.5 Antagonist co-activation level

**Figure 5:**
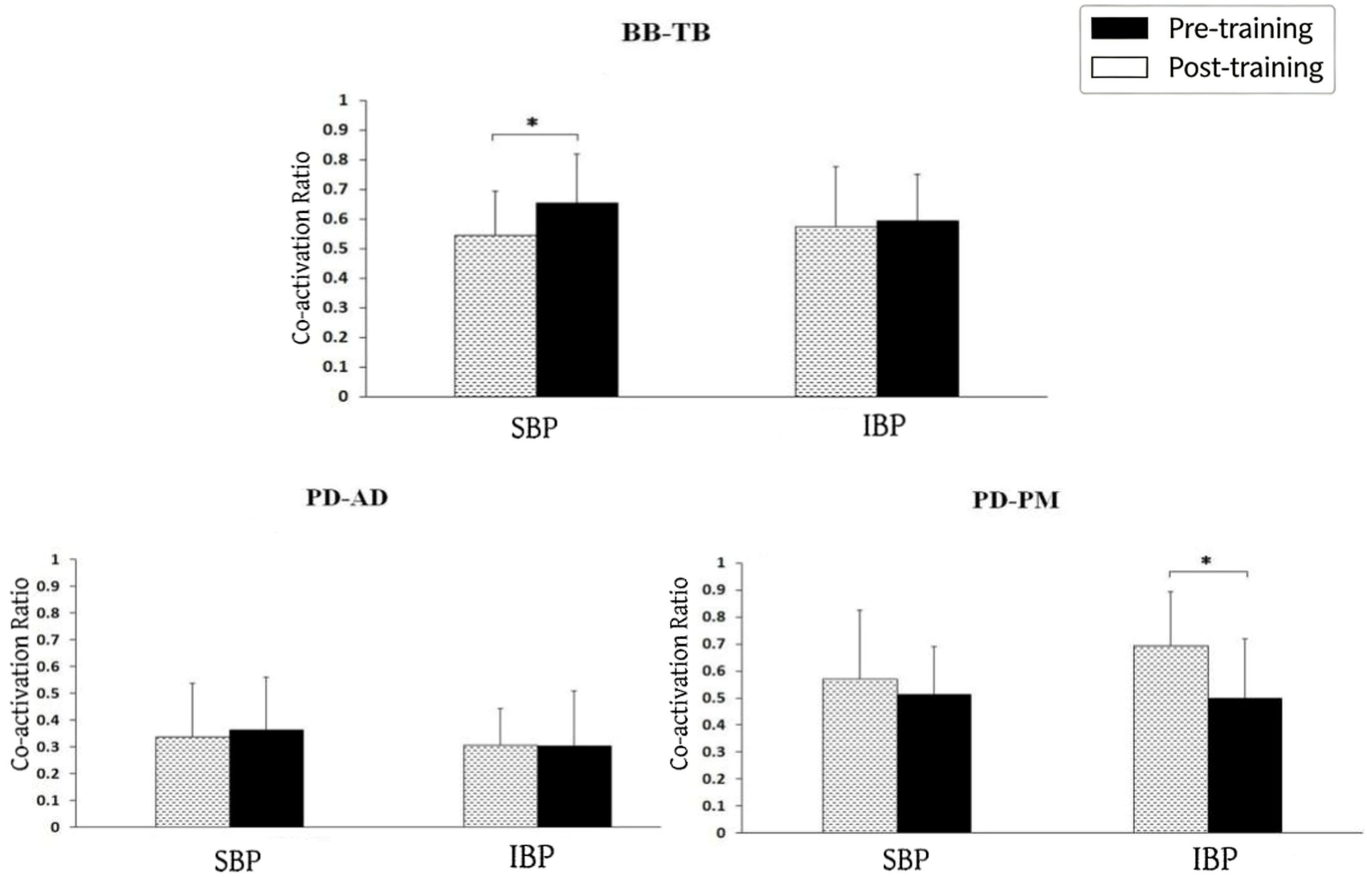
Pre- and post-training comparison of muscle co-activation ratios in IBP test for IBP and SBP groups. IBP, unstable bench press group; SBP, Smith machine bench press group; BB, biceps brachii; TB, triceps brachii; AD, anterior deltoid; PD, posterior deltoid; PM, pectoralis major. *p<0.05 for within-group pre-post comparison.

As shown in **Figure 5**, the results of the bench press barbell test of the IBP group and SBP group under unstable conditions showed that the three pairs of antagonist-agonist muscle groups presented different rules. During the unstable bench press barbell test of the IBP group, the co-activation ratio of BB-TB muscle pair increased significantly compared with before training (P = 0.040), while there was no significant difference in PD-AD and PD-PM (PD-AD: P = 0.600; PD-PM: P = 0.369). After the significant difference test of the co-activation ratio of the IBP group before and after the training intervention, it was found that the co-activation ratio of PD-PM decreased significantly (P = 0.021), while there was no significant change in BB-TB and PD-AD (BB-TB: P = 0.762; PD-AD: P = 0.859).

There was no significant difference in the co-activation ratio of all antagonist-agonist muscle pairs between the two groups (Pre-training: BB-TB: P = 0.736, PD-AD: P = 0.699, PD-PM: P = 0.265; Post-training: BB-TB: P = 0.432, PD-AD: P = 0.699, PD-PM: P = 0.875), indicating that there was no significant difference in the co-activation ratio of each muscle pair between the two groups before and after the training intervention, which was basically at the same level.

### 3.6 Smith machine bench press fatigue test

In the Smith machine bench press fatigue test conducted during the post-training assessment (Kraemer and Ratamess, 2004), the IBP group completed significantly more repetitions than the SBP group (IBP: 15.89 ± 1.90 repetitions; SBP: 10.00 ± 1.23 repetitions; independent t-test: t=7.62, p<0.05, Cohen’s d=1.81, 95% CI = 4.36 to 7.42), suggesting that unstable bench press training may confer additional benefits for muscular endurance in addition to the observed strength gains.

## 4 Discussion

The key findings of this study are as follows: (1) Compared with traditional stable bench press barbell training, 12-week unstable bench press barbell training was associated with greater improvements in dynamic muscle strength (1RM) and movement stability; (2) Unstable bench press training was accompanied by improved activation efficiency of several individual muscles, and significantly reduced co-activation ratio of the PD-PM antagonist muscle pair, suggesting improved intermuscular coordination of muscles.

The positive effect of traditional resistance training on improving muscle strength has been fully confirmed, among which the training method with a load of 65% 1RM and above has a particularly significant effect on the development of maximum muscle strength (Kidgell et al., 2011). For untrained college students, moderate load with standardized movement can effectively stimulate initial strength adaptation. It is well established that loads of 65–75% 1RM, when performed with adequate volume (e.g., 5 sets × 6 repetitions), are sufficient to induce meaningful strength gains in novice populations through neuromuscular adaptation mechanisms including improved motor unit recruitment and firing rate (Kidgell et al., 2011; Radaelli et al., 2015). Unstable conditions increase coordinative difficulty, which compensates for lower nominal intensity. As a classic upper limb strength training movement (del Olmo et al., 2006), bench press is widely used in the daily strength exercise of college students, becoming a commonly used method with good exercise effect in physical education and training related to fitness and bodybuilding, rowing, dragon boat racing and other sports (Gribble et al., 2003). The improvement of muscle strength quality is regulated by multiple factors, among which the neuromuscular regulation and activation ability play a key role in the process of strength development (Osu et al., 2002). Unstable resistance training has been investigated in the context of sports training, with several studies reporting advantages in improving muscle activation efficiency and movement coordination (Lima et al., 2018; Wang et al., 2019; Costello, 2022). However, the broader evidence base remains mixed: some investigations have found that unstable conditions may reduce maximal force output (Behm et al., 2002; Dunnick et al., 2015) or produce neutral strength adaptations compared with stable training (Unhjem et al., 2016; Sanchez-Sanchez et al., 2022). In a study of young soccer players, Sanchez-Sanchez et al (Unhjem et al., 2016). reported that unstable surface training did not provide additional benefits for sprint or jump performance over stable training. Similarly, Zemková et al. (2021) found no cross-transfer effect of unstable resistance training on power output during stable conditions. The present findings, which suggest advantages for unstable training in dynamic strength (1RM) but not in isometric strength (MVIC), may help reconcile these apparent contradictions: the benefits of instability appear to be task-specific and may be most pronounced under testing conditions that share characteristics with the training environment. In the current study, both groups were tested under unstable conditions, which may favor the IBP group and contribute to the observed between-group differences.

On the basis of sorting out the relevant research results of previous scholars on unstable resistance training, combined with the investigation results of the current situation of college students’ strength quality development, this study adopted the unstable bench press training method to carry out a 12-week intervention training for college students, explored its training effect by analyzing the test results of basic physical indicators, electromyography indicators and other indicators, and finally tried to put forward a feasible unstable bench press training scheme suitable for college students.

The results of this study show that the difference in training adaptation effects between the IBP group and the SBP group is mainly attributable to the different regulatory effects of the two training methods on the antagonist co-activation level. The post-training evaluation data show that the antagonist co-activation level of the IBP group decreased significantly compared with before training, while this index of the SBP group had no statistically significant change before and after training. Existing studies have confirmed that the decrease of antagonist co-activation level is closely related to the improvement of agonist-antagonist synergistic control ability and the improvement of neuromuscular efficiency (Palma et al., 2021; Seo et al., 2022; Sousa et al., 2022; Christian et al., 2023; Clark, 2024). It is plausible that in dynamic pushing movements, unstable resistance training (IBP) may be more conducive to improving muscle contraction efficiency than stable resistance training (SBP).

Motor learning research suggests that the nervous system can develop an “internal model” of movement dynamics through repeated practice, refining coordination strategies to improve movement efficiency (Chulvi-Medrano et al., 2010; Franchi et al., 2018; Lima et al., 2018). In this study, the decrease range of the co-activation level of biceps brachii and middle deltoid in the IBP group was significantly larger than that in the SBP group, which is consistent with previous findings on neuromuscular adaptation to unstable training (Wang et al., 2022). The above finding suggest that unstable training conditions may facilitate more efficient neuromuscular coordination, potentially by reducing excessive antagonist activation during the concentric phase of the bench press movement.

Neuromuscular control and movement stability are inherently interdependent (Unhjem et al., 2016; Lim et al., 2022; Sanchez-Sanchez et al., 2022). The decrease of neuromuscular control ability will directly lead to the decrease of joint stability, thus increasing the dependence on antagonist co-activation to compensate for mechanical defects (Domeika et al., 2018; Moosaei et al., 2024); on the contrary, a scientifically designed training program can optimize the neural coordination mode, thus improving joint stability and reducing unnecessary muscle co-activation (Granacher et al., 2013; Eckardt and Rosenblatt, 2019). The results of this study show that the movement acceleration of the IBP group during bench press is significantly reduced, which index directly reflects the improvement of its movement stability (Arnold and Bautmans, 2014; Biebl et al., 2022). This adaptive improvement is particularly relevant for college students, as enhanced movement control may contribute to safer and more effective execution of resistance exercises in physical education settings. According to the principle of exercise specificity (Cohen, 1992; Thomas et al., 2011; Miller, 2012), the stability adaptations acquired through unstable resistance training are most likely to transfer to activities that share similar neuromuscular coordination and muscle activation demands. However, it should be acknowledged that the testing was conducted under unstable conditions, which may provide a task-specific advantage to the IBP group, and the degree of transfer to stable performance contexts warrants further investigation.

From the neuromuscular level, the RMS amplitude of antagonist electromyography in the IBP group was significantly lower than that in the SBP group, which result further supports the interpretation that the improvement of movement stability may be derived from the enhancement of neuromuscular coordination ability. Taken together, the instability stimulation introduced by elastic bands appears to promote adaptations in neuromuscular coordination, enhancing agonist muscle efficiency while reducing unnecessary antagonist co-activation. These adaptations likely reflect improved intermuscular coordination rather than direct structural reorganization of the central nervous system, as no direct CNS measures were collected in this study. This unstable training device is low-cost, easy to install, highly adjustable, space−saving, and suitable for college physical education environments. It is worth noting that the training program adopted in this study has strong practicability and replicability, which is convenient for coaches to integrate unstable training system into resistance training programs, and realize the optimization and improvement of exercise efficiency by synchronously improving the joint stability and neuromuscular regulation ability of trainers.

Several limitations of this study should be acknowledged. First, all participants received the same 2-week unstable familiarization training, and all biomechanical and neuromuscular tests were conducted under unstable conditions. This design introduces a potential task-specific testing bias that may favor the unstable training group (IBP), making it difficult to fully disentangle true training adaptations from testing familiarity effects. Although this approach ensured standardization and safety in untrained participants, future studies should consider including both stable and unstable testing conditions to isolate training-specific effects from testing-specific advantages. Second, only male college students were included as research subjects, and the potential gender-related effects may reduce the consistency and universality of the research results. Third, in the bench press test, to minimize the impact of rhythm differences on the performance of maximum repetitions and muscle activation, we controlled the bench press rhythm of the subjects. However, this standardized rhythm may vary due to the different familiarity and adaptability of individuals to the applied rhythm. Fourth, in this study, only five major muscles were collected for testing and analysis according to previous studies (Radaelli et al., 2015), while other muscles (such as trapezius and serratus anterior) were not tested and analyzed. Fifth, using only arm circumference to reflect the muscle hypertrophy caused by training has limitations in accuracy and specificity (Schoenfeld et al., 2017; Roberts et al., 2020). Sixth, crosstalk is still an inherent problem in electromyographic activity assessment (Behm et al., 2002). Although the electrode placement is strictly standardized and performed by experienced researchers to reduce the impact of crosstalk, the impact of crosstalk on the results still cannot be ignored. Seventh, the two training interventions employed different nominal loading schemes (IBP: 55–75% 1RM; SBP: 65–85% 1RM), which, although physiologically justified, precludes a load-matched between-group comparison. The differential loading should be considered when interpreting the relative efficacy of each training modality. Eighth, the untrained status of the participants and the use of the Smith machine rather than a free barbell may limit the generalizability of the findings to athletic or trained populations and to free-weight bench press performance.

## 5 Conclusion

### 5.1 Research conclusions and recommendations

Unstable bench press training was associated with improvements in agonist muscle activation efficiency and intermuscular coordination, as reflected by reduced antagonist co-activation during the concentric phase. Compared with the Smith machine bench press training, the unstable training group demonstrated greater gains in dynamic maximal strength (1RM) and movement stability, although the interpretation of these differences should be tempered by the task-specific nature of the testing procedures, which were conducted under unstable conditions. These findings are consistent with the hypothesis that local instability introduced through elastic bands can enhance neuromuscular demand without substantially compromising force output—unlike traditional global instability approaches that often result in neutral or negative strength adaptations. The present results should be viewed as preliminary evidence that unstable bench press training may serve as a useful adjunct to conventional resistance training for improving upper limb strength and movement control in untrained college students. Further research incorporating both stable and unstable testing conditions, load-matched designs, and direct measures of neuromuscular function is warranted to confirm and extend these findings.

For unstable bench press barbell training, the agonist contraction ability and muscle contraction efficiency (antagonist co-activation ratio) showed significant improvements after the 12-week intervention, which may reflect adaptations in neuromuscular coordination and intermuscular regulation. This suggests that unstable bench press training may improve the activation efficiency of individual muscles and significantly reduce the co-activation ratio of specific antagonist muscle pairs, thus improve the synergistic working ability between muscles.

## Data Availability

The original contributions presented in the study are included in the article/Supplementary Material. Further inquiries can be directed to the corresponding author.
